# Establishment and validation of a novel invasion-related gene signature for predicting the prognosis of ovarian cancer

**DOI:** 10.1186/s12935-022-02502-4

**Published:** 2022-03-15

**Authors:** Leilei Liang, Jian Li, Jing Yu, Jing Liu, Lin Xiu, Jia Zeng, Tiantian Wang, Ning Li, Lingying Wu

**Affiliations:** Department of Gynecologic Oncology, National Cancer Center/National Clinical Research Center for Cancer/Cancer Hospital, Chinese Academy of Medical Sciences and Peking Union Medical College, Beijing, 100021 China

**Keywords:** Ovarian cancer, Invasion, 6-gene signature, Risk, Prognosis

## Abstract

**Background:**

Ovarian cancer (OC) is an invasive gynaecologic cancer with a high cancer-related death rate. The purpose of this study was to establish an invasion-related multigene signature to predict the prognostic risk of OC.

**Methods:**

We extracted 97 invasion-related genes from The Cancer Genome Atlas (TCGA) database. Then, the ConsensusClusterPlus and limma packages were used to calculate differentially expressed genes (DEGs). To calculate the immune scores of the molecular subtypes, we used ESTIMATE to evaluate the stromal score, immune score and ESTIMATE score. MCP-counter and the GSVA package ssgsea were used to evaluate the types of infiltrating immune cells. Survival and nomogram analyses were performed to explore the prognostic value of the signature. Finally, qPCR, immunohistochemistry staining and functional assays were used to evaluate the expression and biological abilities of the signature genes in OC.

**Results:**

Based on the consistent clustering of invasion-related genes, cases in the OC datasets were divided into two subtypes. A significant difference was observed in prognosis between the two subtypes. Most genes were highly expressed in the C1 group. Based on the C1 group genes, we constructed an invasion-related 6-gene prognostic risk model. Furthermore, to verify the signature, we used the TCGA-test and GSE32062 and GSE17260 chip datasets for testing and finally obtained a good risk prediction effect in those datasets. Moreover, the results of the qPCR and immunohistochemistry staining assays revealed that KIF26B, VSIG4 and COL6A6 were upregulated and that FOXJ1, MXRA5 and CXCL9 were downregulated in OC tissues. The functional study showed that the expression of KIF26B, VSIG4, COL6A6, FOXJ1, MXRA5 and CXCL9 can regulate the migration and invasion abilities of OC cells.

**Conclusion:**

We developed a 6-gene prognostic stratification system (FOXJ1, MXRA5, KIF26B, VSIG4, CXCL9 and COL6A6) that is independent of clinical features. These results suggest that the signature could potentially be used to evaluate the prognostic risk of OC patients.

**Supplementary Information:**

The online version contains supplementary material available at 10.1186/s12935-022-02502-4.

## Introduction

Despite advances in the diagnosis and treatment of ovarian cancer (OC) over the past few decades, long-term survival remains poor [1, 2]. OC is a malignant gynaecologic cancer and is the eighth leading cause of cancer-related death worldwide [3]. The standard treatment of OC includes surgery and platinum-based chemotherapy. Currently, the 5-year survival rate of OC is approximately 47%, which is primarily due to recurrence and chemical resistance [4]. Emerging therapies for high-grade serous ovarian carcinoma include molecular targeting agents [5–8], including PARP inhibitors. The results of 4 phase III clinical trials (SOLO-1, PAOLA-1/ENGOT-OV25, PRIMA/ENGOT-OV26 and VELIA/GOG-3005) showed that PARP inhibitors can significantly improve the progression-free survival of OC patients [9]. OC is mainly diagnosed by transvaginal ultrasonography and blood CA-125 levels, but these methods have limitations. Transvaginal ultrasound can result in the misdiagnosis of cancer as cysts, and blood CA-125 levels have a high false-positive rate [10–12]. The current low accuracy in the diagnosis of early OC highlights the demand for new diagnostic biomarkers.

Tumour biomarkers play a crucial role in the detection and management of OC. Many oncogenes or suppressor genes associated with OC have been reported, including noncoding RNAs and mRNAs [13, 14]. Previous studies have shown that the inhibition of HDAC6 gene expression reduces the proliferation, migration and survival of OC cells [15]. It has also been shown that the downregulation of RAD51AP1 inhibits the proliferation, migration and invasion of OC cells in vitro [16]. BRDT overexpression promotes the development of OC cells [17]. Although many genes have been found to be associated with the diagnosis and prognosis of OC, gene markers for diagnosis and prognosis should still be studied.

In this study, tumour invasion-related genes were collected, and crossover analysis with gene expression datasets from The Cancer Genome Atlas (TCGA) and Gene Expression Omnibus (GEO) databases was performed. We established OC invasion-related molecular subtypes. The relationships between molecular subtype and prognosis and clinical features were further evaluated. The prognostic risk model constructed using differentially expressed genes (DEGs) between OC molecular subtypes can better evaluate the prognosis of OC patients, and the GEO gene expression dataset was further used to verify the prognostic risk model. Moreover, the results of qPCR and immunohistochemistry (IHC) staining assays revealed that KIF26B, VSIG4 and COL6A6 were upregulated and that FOXJ1, MXRA5 and CXCL9 were downregulated in OC tissues. The functional study showed that the expression of KIF26B, VSIG4, COL6A6, FOXJ1, MXRA5 and CXCL9 can regulate the migration and invasion abilities of OC cells. These results suggest that the signature could potentially be used to evaluate the prognostic risk of OC patients.

## Methods

### Data download

We downloaded the most recent expression data and clinical follow-up information of OC patients in the TCGA database, which contains RNA-Seq samples along with gene expression profile information. The GSE32062 and GSE17260 chip datasets with prognostic information were downloaded from the GEO database. Invasion-related gene sets, which included 97 genes (Additional file 4: Table S1), were downloaded from CancerSEA [18].

### Comparative analysis of immune scores between molecular subtypes

To identify the relationship between the immune scores of molecular subtypes in the TCGA dataset, we used the R software package ESTIMATE to evaluate the stromal score, immune score and ESTIMATE score; MCP-counter was used to evaluate 10 types of infiltrating immune cells, and the GSVA package ssgsea was used to evaluate 28 types of infiltrating immune cells [19]. We then compared the differences in immune scores between the molecular subtypes. Next, according to a previous report [20], TCGA-OC samples were classified according to mRNA expression and divided into four categories: differentiated, immunoreactive, mesenchymal and proliferative. Finally, we compared the relationship between C1 and C2 obtained by our classification.

### Construction of a prognostic risk model

First, 363 samples in the TCGA dataset were divided into a training set and a test set. To avoid the influence of random allocation deviation on the stability of subsequent modelling, all samples were randomly grouped 100 times in advance, and grouping was performed according to a 1:1 ratio of the training set and test set. Using the training set data, a univariate Cox proportional hazards regression model was performed using the R package survival with the coxph function; p  < 0.05 was selected as the threshold for filtering. According to previously identified DEGs, we used the R software package glmnet for least absolute shrinkage and selection operator (lasso) Cox regression to further compress the screened genes and to reduce the number of genes in the risk model. Stepwise regression uses the pooled Akaike information criterion (AIC), which considers the statistical fitting degree of the model and the number of parameters used for the fit. The step method in the stats package starts from the most complex model and deletes a variable in turn to reduce the AIC. This shows that the model exhibits a sufficient degree of fit with fewer parameters.

### Specimen collection

Ovarian tumour and normal tissues derived from surgical resection specimens were snap-frozen in liquid nitrogen and stored at − 80 °C until RNA extraction. The clinicopathological characteristics of ovarian cancer tissues shown in Additional file 5: Table S2. None of the patients received chemotherapy or radiation therapy. None of the patients received treatment before surgery, and all patients signed informed consent forms provided by the Cancer Hospital, CAMS & PUMC. This study was approved by the Ethics Committee of the Cancer Institute (Hospital), CAMS & PUMC (17-099/1355). Ten ovarian tumour tissues were high-grade serous adenocarcinoma.

### Cell culture and treatments

The human OC cell line SKOV3 (Cat: HTB-7) cells were purchased from American Type Culture Collection (ATCC). SKOV3 is an ovarian cancer cell line that was established from an Ovarian adenocarcinoma tumour in an untreated patient. A2780 (93,112,519) cell lines were purchased from the European Collection of Authenticated Cell Cultures (ECACC, Sigma-Aldrich, St. Louis, MO, USA). The human OC cell line A2780 is an ovarian cancer cell line that was established from an Ovarian endometroid adenocarcinoma tumour in an untreated patient. The cell line has an epithelial morphology and cells grow as a monolayer in tissue culture flasks and in suspension in spinner cultures. The patient from whom the SKOV3 and A2780 cell line was established, did not receive treatment for their tumour before tissue was taken. The cell lines were cultured in DMEM supplemented with 10% foetal bovine serum (Invitrogen, San Diego, CA) in a 5% CO_2_ incubator at 37 °C. Si-MXRA5, Si-KIF26B, Si-VSIG4, Si-COL6A6 and Si-NC were purchased from GenePharma (Shanghai, China). The human FOXJ1 and CXCL9 coding sequences were purchased from Fenghui (Hunan, China). Transfection was performed using Lipofectamine 3000 reagent (No. L3000015, Invitrogen, China) according to the manufacturer’s instructions. The sequences are shown in Additional file 6: Table S3.

### Total RNA extraction and quantitative real-time PCR

Total RNA was extracted from 10 ovarian tumour and 6 nontumour tissues using RNA-easy Isolation Reagent (No. RC112-01, Vazyme, China). Quantitative real-time PCR (qRT-PCR) analysis was performed using the HiScript III 1st Strand cDNA Synthesis Kit (No. R312-01, Vazyme, China) and ChamQTM Universal SYBR^®^ qPCR Master Mix (No. Q712-02, Vazyme, China) according to the manufacturer’s instructions. The primers are shown in Additional file 6: Table S3. GAPDH served as an internal control.

### IHC staining analysis

An immunohistochemistry SP kit (No. SP-9000, ZSGB-BIO, China) was used for IHC, which was performed as previously described [21]. Anti-FOXJ1 (1:200) and anti-MXRA5 (1:200) were purchased from Immunoway (No. YT1751; No. YN2103, USA); anti-KIF26B (1:200) and anti-VSIG4 (1:200) were purchased from Abcam (No. ab121952; No. ab252933, China); anti-CXCL9 (1:200) was purchased from Affinity (No. DF9920, China); and anti-COL6A6 (1:200) was purchased from Invitrogen (No. PA5-60958, China). The magnification of the IHC images was 20 ×.

### Transwell assays

Transwell assays were performed as previously described [22]. Transwell assays were used to determine the invasion and migration abilities of CAOV3 and A2780 cells in vitro. For the migration assay, 700 μl DMEM with 20% serum was added to the lower chamber of a Transwell plate (Corning, NY, USA), and 1.5 × 105 cells were added to 200 μl serum-free DMEM in the upper chamber. After incubation for 24 h at 37 °C, the Transwell chamber was removed, cleaned once with PBS, and fxed for 30 min, then stained with 0.5% crystal violet for 30 min. The chamber was rinsed with PBS, and the cells on the upper side of the flter were carefully wiped away.

### Statistical analysis

All statistical analyses were performed with R software 3.5.3 and GraphPad Prism v. 8.01 (GraphPad Software, La Jolla, CA, USA). Student’s t test was used to compare values between the test and control groups. p values < 0.05 indicated statistical significance.

## Results

### Identification of two molecular subtypes based on invasion-related gene profiles

**Fig. 1:**
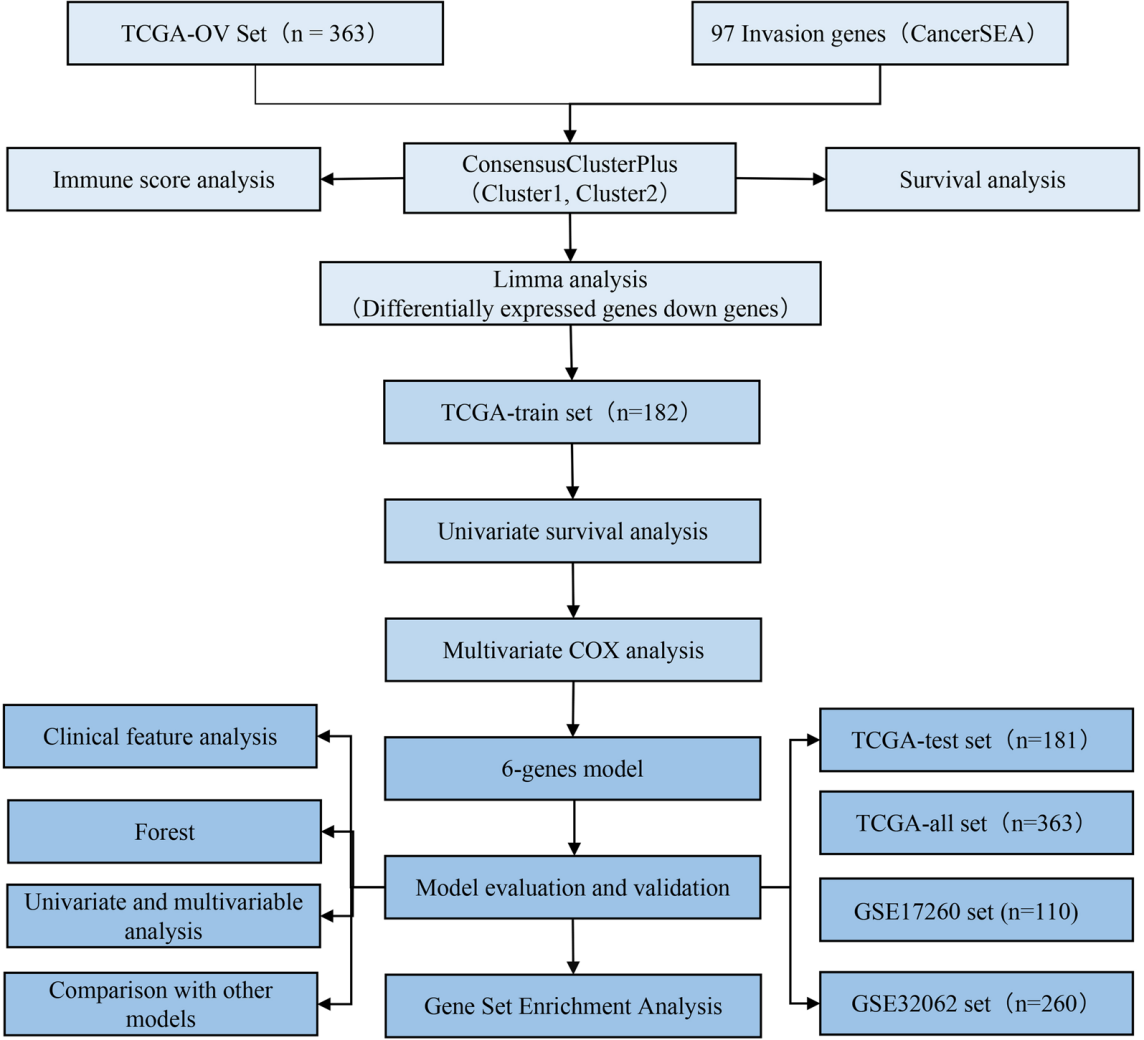
Technical road map

**Fig. 2:**
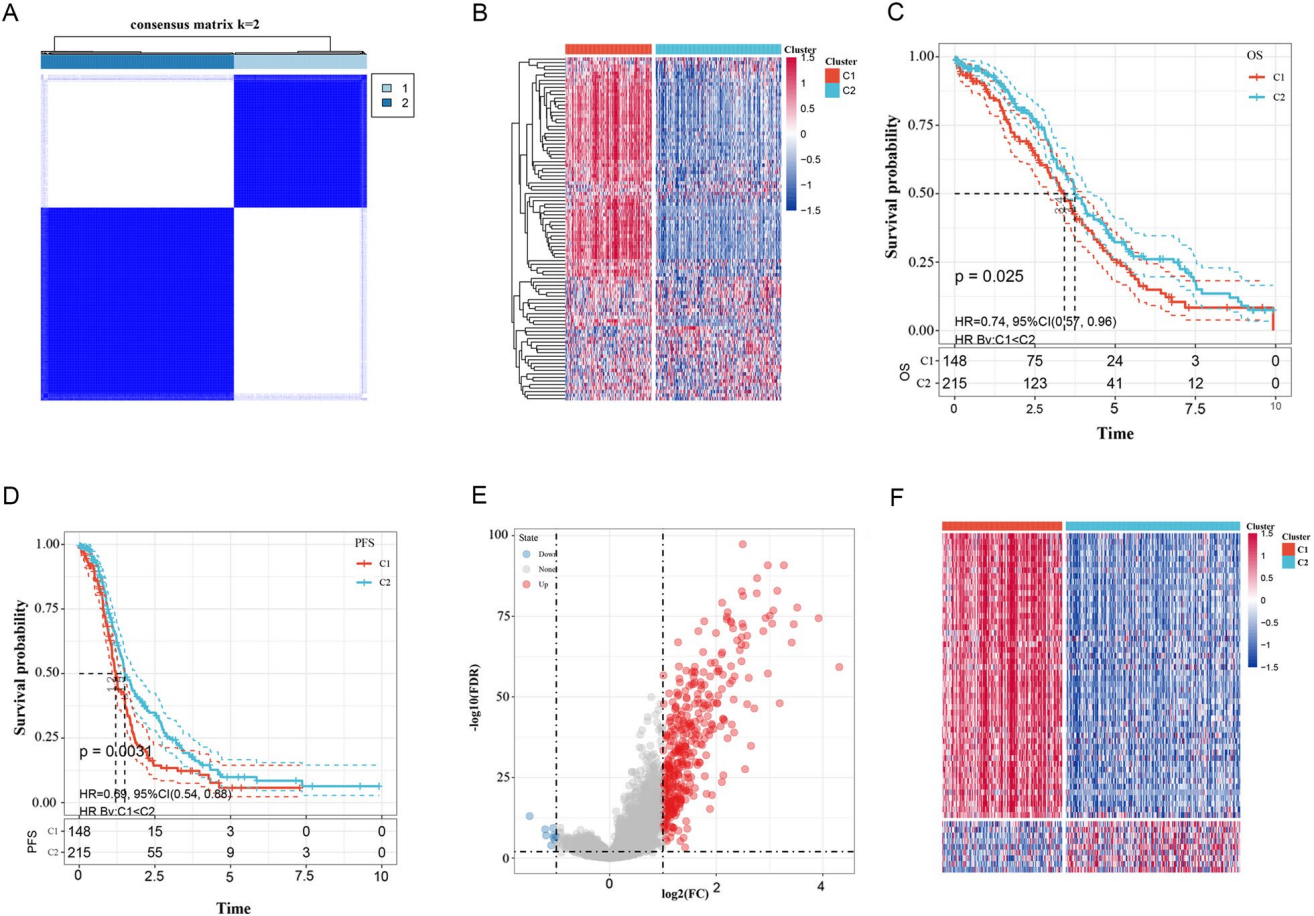
Sample clustering heat map (**A**); heat map of 97 invasion-related genes (**B**); OS of each molecular subtype (**C**); PFS of each molecular subtype (**D**); volcano maps (**E**) and heat map (**F**) of differentially expressed genes between the C1 and C2 groups;

After preprocessing, the TCGA-OC dataset had 363 samples, the GSE32062 dataset had 260 samples, and the GSE17260 dataset had 110 samples (Fig. 1). The clinical statistics of the samples are shown in Table 1. We first extracted the expression of 97 invasion-related genes from the TCGA database and then used ConsensusClusterPlus to cluster these genes. At k = 2, the samples could be clustered together (Fig. 2A). The expression of 97 invasion-related genes in the two subclasses is shown in Fig. 2B; most genes were highly expressed in the C1 group and expressed at low levels in the C2 group. We further analysed the prognostic relationship between the two groups, and the results revealed significant differences between C1 and C2 (Fig. 2C, D; p < 0.05). DEGs between the C1 and C2 molecular subtypes were determined by the limma package. In total, 393 DEGs were filtered according to the thresholds of false discovery rate (FDR) < 0.05 and |log_2_ fold change (FC)|> 1. There were 384 upregulated genes and 9 downregulated genes (Fig. 2E and Additional file 7: Table S4). We selected the top 50 DEGs to generate a heat map (Fig. 2F).

**Table 1:** Sample information table

Clinical features	TCGA-OC	GSE32062	GSE17260
OS			
0	136	139	64
1	227	121	46
Stage			
I	1		
II	21		
III	282		
IV	56		
X	3		
Grade			
G1	1		
G2	42		
G3	310		
G4	1		
GX	10		
Age			
≤ 60	199		
> 60	164

Furthermore, we performed Kyoto Encyclopedia of Genes and Genomes (KEGG) pathway and Gene Ontology (GO) functional enrichment analyses on the DEGs. For GO functional annotation, the results of the top 10 biological processes (BPs), molecular functions (MFs) and cellular components (CCs) are shown in Additional file 1: Figure S1A–C. For KEGG pathway enrichment, the annotation results are shown in Additional file 1: Figure S1D, in which ECM-receptor interaction, proteoglycans in cancer, focal adhesion, PI3K-Akt signalling pathway, and other tumour-related pathways were significant. More detailed information is shown in Additional file 8: Table S5.

Next, we used gene set enrichment analysis (GSEA) to analyse the pathways that were significantly enriched in the C1 and C2 groups. The thresholds for enriched pathways were p < 0.05 and FDR < 0.25; we then obtained the significantly enriched pathways (Additional file 9: Table S6). More tumour-related pathways were enriched in the C1 subgroup, such as APOPTOSIS, PATHWAYS_IN_CANCER, VEGF_SIGNALING_PATHWAY, TOLL_LIKE_RECEPTOR_SIGNALING_PATHWAY, ECM_RECEPTOR_INTERACTION, FOCAL_ADHESION and other tumour-related pathways. These GSEA pathways are shown in Additional file 1: Figure S1E and indicate that the C1 subtype was more strongly correlated with tumours.

### Comparative analysis of immune scores between the two molecular subtypes

**Fig. 3:**
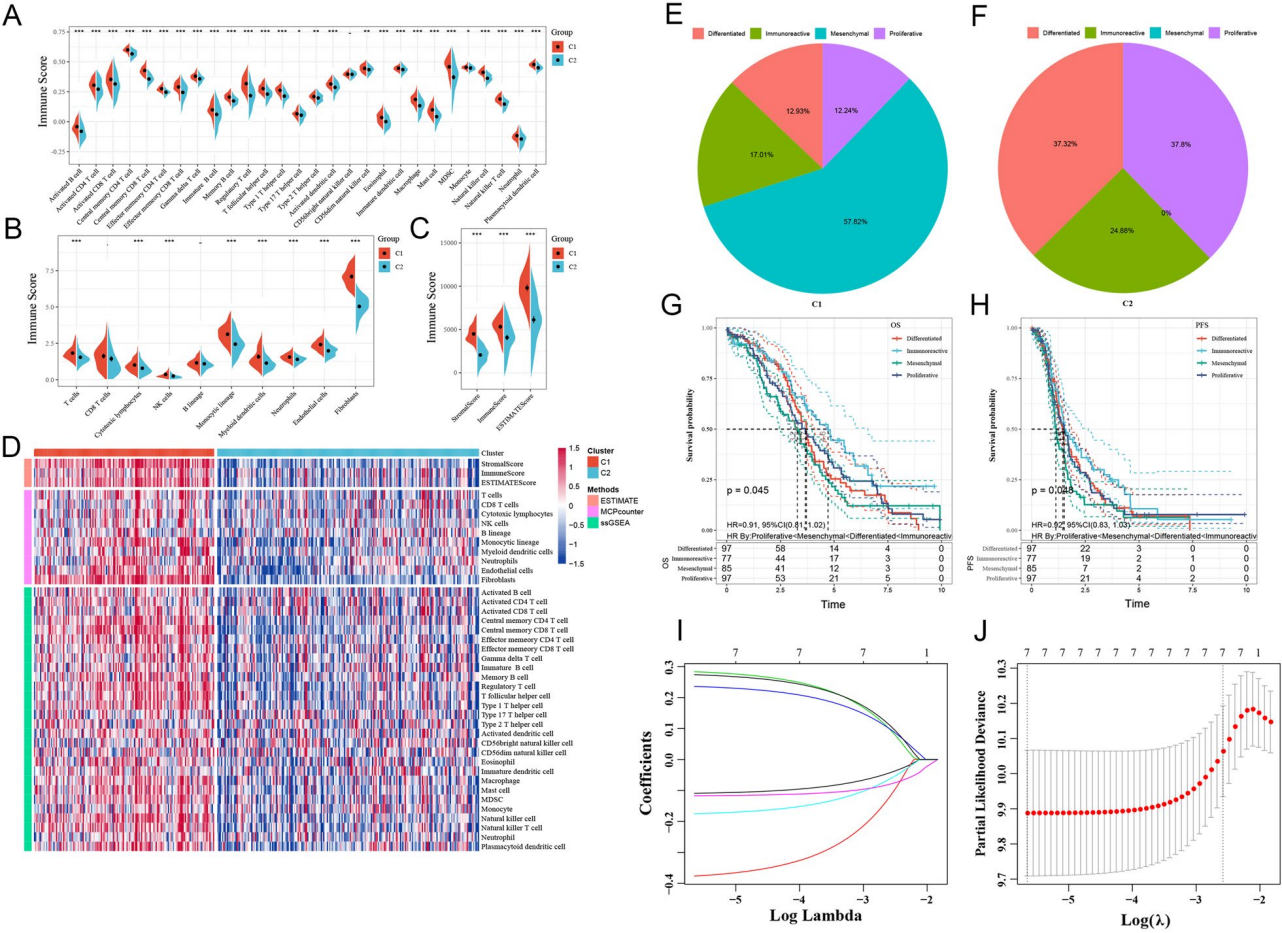
Comparison of ssGSEA (**A**), MCP-counter (**B**) and estimated immune scores between molecular subtypes (**C**) and a heatmap of the immune score results (**D**) in the TCGA dataset. Comparison between molecular subtype C1 and existing subtypes (**E**); **B** comparison between molecular subtype C2 and existing subtype C (**F**); OS of existing molecular subtypes (**G**); **D** PFS of existing molecular subtypes (**H**). The trajectory of each independent variable: the horizontal axis represents the log value of the independent variable lambda, and the vertical axis represents the coefficient of the independent variable (**I**). The confidence interval is under each lambda (**J**)

ESTIMATE was used to identify the relationship between the immune scores of the molecular subtypes. The results showed that the immune score of the C1 subtype was higher than that of the C2 subtype (Fig. 3A–C). We also generated a heatmap of the immune scores of the two subtypes (Fig. 3D).

### Comparison of the identified molecular subtypes and existing subtypes

According to a previous report [13], the TCGA-OC samples were classified based on mRNA expression, after which they were divided into four categories: differentiation, immunoreactive, mesenchymal and proliferative. We compared the relationship between the C1 and C2 subtypes obtained by our classification and found that the proportion of mesenchymal cells in the C1 subtype obtained by our clustering reached 57.82% (Fig. 3E). The proportion of proliferative cells in the C2 subtype obtained by our clustering reached 37.8% (Fig. 3F). Furthermore, we analysed the prognostic relationships among these four subtypes and found significant differences in differentiation, immunoreactivity, mesenchymal activity and proliferation (Fig. 3G, H; p < 0.05).

### Construction and evaluation of a prognostic risk model

The training set data were used for further analyses. Univariate Cox analysis was performed on 393 DEGs among molecular subtypes, and 7 genes that were associated with prognosis were identified (Additional file 10: Table S7). Lasso regression was used to reduce the gene numbers in the risk model. First, we analysed the trajectory of each independent variable, as shown in Fig. 3I. Next, we used ten-fold cross validation to construct the model and confidence interval under each lambda, as shown in Fig. 3J. From the graph, we chose 7 genes (FOXJ1, MXRA5, KIF26B, VSIG4, CXCL9, CCL19 and COL6A6) when lambda was 0.003518527. Furthermore, a stepwise regression algorithm was applied, and we finally reduced 7 genes to 6 genes, namely, FOXJ1, MXRA5, KIF26B, VSIG4, CXCL9 and COL6A6. The prognostic Kaplan–Meier (KM) curves of the 6 genes are shown in Additional file 1: Figure S1F. The final 6-gene signature formula is as follows:

Riskscore = − 0.1120494*FOXJ − 0.3964107*MXRA5 + 0.2782800*KIF26B + 0.2701755*VSIG4 − 0.2419422*CXCL9 + 0.2652037*COL6A6.

### Verification of the prognostic risk model

**Fig. 4:**
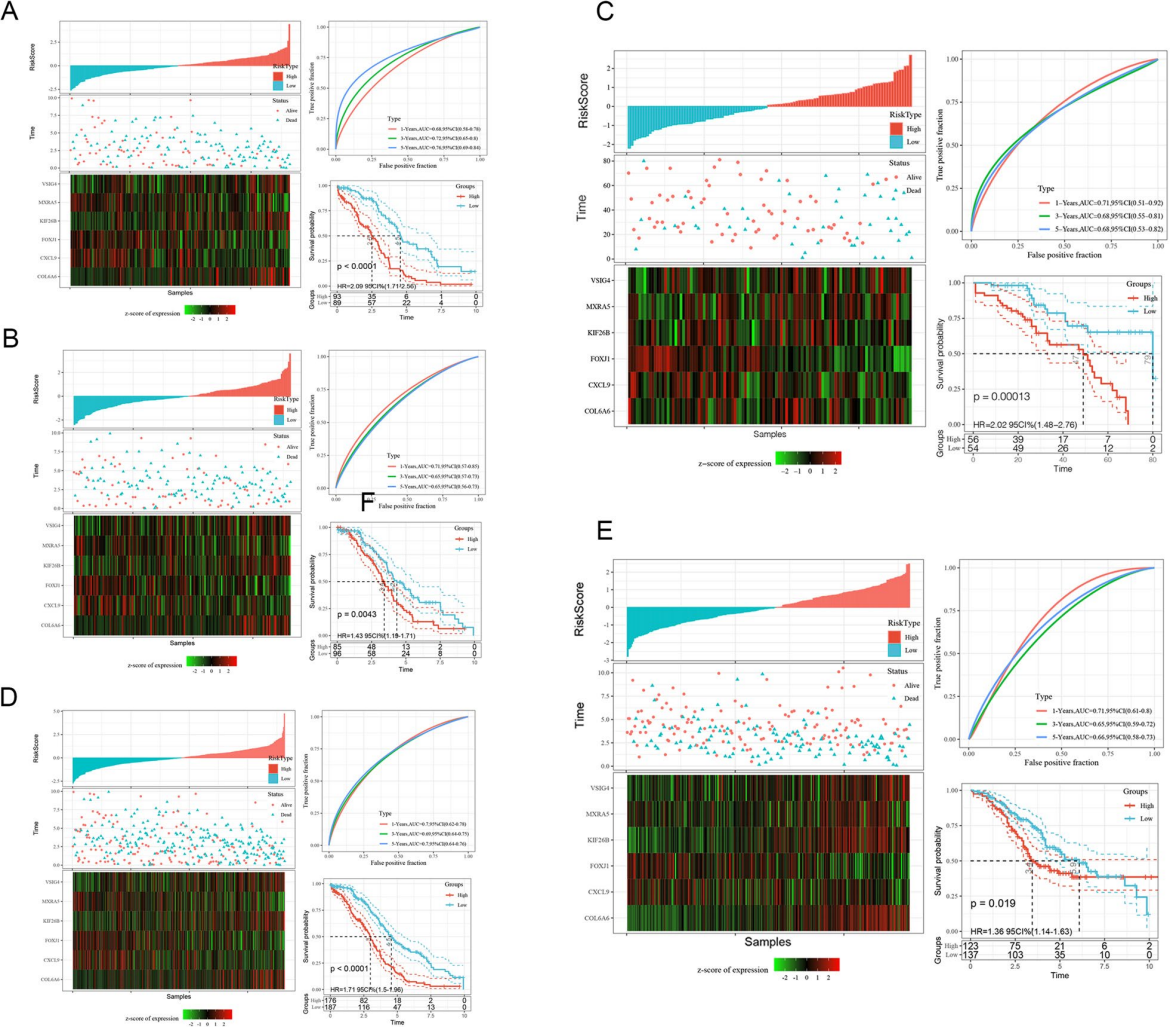
Risk score, survival time, survival status, and 6-gene signature expression in the TCGA training set. ROC curve and AUC of the 6-gene signature classification; 6-gene signature distribution within the KM survival curve in the training set (**A**). TCGA test set risk score, survival time, survival status and 6-gene signature expression (**B**); risk score, survival time, survival status, and 6-gene signature expression in all TCGA datasets (**C**). Independent test of the risk score, survival time, survival status, and expression of the 6-gene signature in GSE32062 (**D**). Independent test of the risk score, survival time, survival status, and 6-gene signature expression in GSE17260 (**E**)

We calculated the risk score of the TCGA training set and drew the risk score distribution. Based on the graph, samples with a high risk score were significantly lower in the graph than those with a low risk score, which suggests that high-risk score samples had a worse prognosis. The changes in expression of the 6 genes in the signature with increasing risk value were identified. High expression of KIF26B, VSIG4 and COL6A6 was a risk factor, while high expression of FOXJ1, MXRA5 and CXCL9 was a protective factor. Furthermore, we used the R software package timeROC to analyse the receiver operating characteristic (ROC) curve of the risk score. We analysed the classification efficiency of the 1-year, 3-year and 5-year prognostic predictions. Finally, we calculated the z-scores of the risk score, divided the samples with a risk score greater than zero after the z-score into a high-risk group and those with a risk score less than zero into a low-risk group, and drew KM curves, as shown in Fig. 4A. The high-risk group had a lower survival rate (p < 0.0001).

To determine the robustness of the model, we utilized the TCGA test set and all TCGA datasets to calculate the risk score and drew the risk score distribution. Samples with a high risk score were significantly lower on the graph than those with a low risk score. The results of the KM curves reveal that the high-risk group has a lower survival rate in the TCGA test set and all TCGA datasets (Fig. 4B, C) (p < 0.01).

We used the same model and coefficients as those in the training set in the external test sets GSE32062 and GSE17260. We also calculated the risk score of each sample according to the expression level of the sample and drew the risk score distribution of the sample. The risk score distributions of the independent test datasets GSE32062 and GSE17260 are shown in Fig. 4D, E.

### Prognostic analysis of the risk model and clinical features

We further performed correlation analysis of the 6-gene signature and clinical factors and found that the signature could significantly distinguish high- and low-risk groups by age, stage III, stage IV, grade, recurrence or chemotherapy; however, stage I + II and no chemotherapy could not be used to distinguish between high- and low-risk groups due to the limited number of samples (Additional file 2: Figure S2A; p < 0.05). This finding further shows that our model still has good predictive ability for some different clinical factors.

### Performance of the risk score for different clinical features and molecular subtypes

**Fig. 5:**
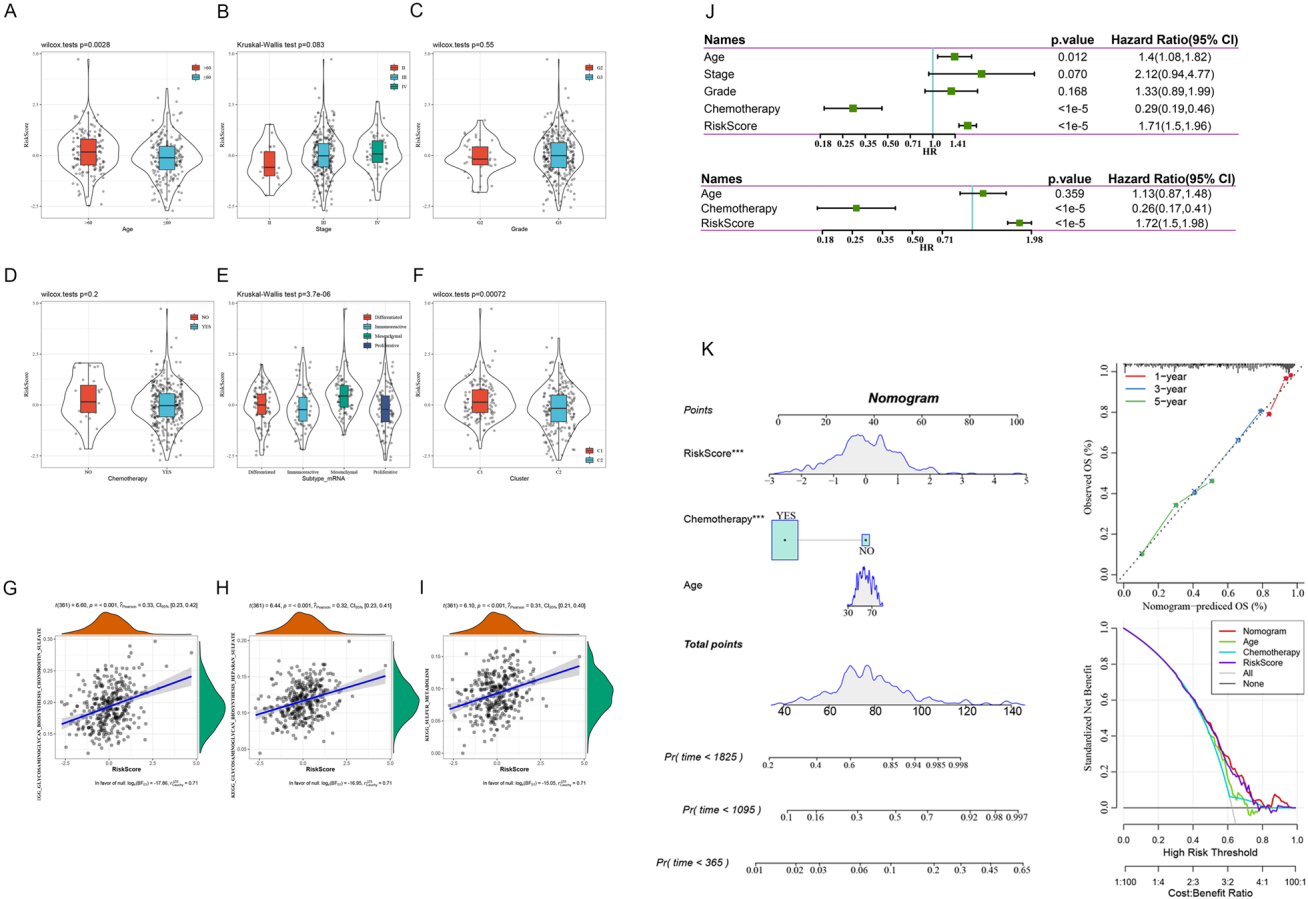
By comparing the distribution of the risk score among clinical feature groups, significant differences were found among age groups, subtypes, and clusters (**A**, **E**, **F**); no significant difference was found among the stage, grade and chemotherapy groups (**B**–**D**); correlation coefficient between KEGG pathways and the risk score with a risk score greater than 0.3 (**G**–**I**); single-factor (**J**) and multivariate analysis results of the clinical characteristics and the risk score. Construction and evaluation of the line graph model (**K**)

By comparing the distribution of the risk score among different clinical feature groups, we found significant differences among age groups, subtypes, and clusters (Fig. 5A, E, F; p < 0.05) and no significant differences among the stage, grade and chemotherapy groups (Fig. 5B–D; p > 0.05). The results showed that for our molecular subtypes, the risk score of the C1 subtype with a poor prognosis was significantly higher than that of the C2 subtype with a good prognosis. Significant differences were also found in the risk score between existing molecular subtypes.

### Relationship between the risk score and pathways

To observe the relationship between the risk score and the biological functions of different samples, the gene expression profiles corresponding to these samples were selected for single-sample GSEA using the R software package GSVA. The scores of each sample for different functions were calculated to obtain the ssGSEA scores of each function corresponding to each sample. The correlations between these functions and the risk score were further calculated. The functions with correlations greater than 0.3 were selected, as shown in Fig. 5G–I. The KEGG_GLYCOSAMINOGLYCAN_BIOSYNTHESIS_CHONDROITIN_SULFATE, KEGG_GLYCOSAMINOGLYCAN_BIOSYNTHESIS_HEPARAN_SULFATE, and KEGG_SULFUR_METABOLISM pathways were positively correlated with the risk scores of the samples.

### Univariate and multivariate analyses of the 6-gene signature

To assess the independence of the 6-gene signature model for clinical application, we used single-factor and multifactor Cox regression to analyse the clinical information and the risk score. In the TCGA dataset, univariate Cox regression analysis showed that the risk score was significantly associated with survival, and multivariate Cox regression analysis showed that the risk score (HR = 1.72, 95% CI 1.5–1.98, p < 1e−5) was still significantly associated with prognosis Fig. 5J. The above findings show that our 6-gene signature model has good predictive performance in clinical application.

### Construction of a nomogram integrating the risk score and clinical features

According to the results of univariate and multivariate analyses, we constructed a nomogram model with clinical features (age, chemotherapy and risk score) (Fig. 5K). In the model, the risk score features have the greatest impact on survival prediction, which indicates that the 6-gene risk model can better predict prognosis. Furthermore, we used a calibration curve to evaluate the prediction accuracy of the model. The prediction calibration curves of the three calibration points at 1, 3 and 5 years are close to the standard curve, which indicates that the model has good prediction performance. In addition, we used decision curve analysis (DCA) to evaluate the reliability of the model. We observed that the benefits of the risk score and nomogram were significantly higher than the extreme curve and that the benefits of the nomogram were higher than those of the risk score. Age and chemotherapy were close to the extreme curve, which indicates that the risk score and nomogram have good reliability.

### Model comparison and immunotherapy cohort prediction

We finally selected two prognostic risk models, a 10-gene signature (Wang) [23] and a 7-gene signature (Sabatier) [24], for comparison with our 6-gene signature model. To make the models comparable, we calculated the risk score of each OC sample in the TCGA dataset using the same method based on the corresponding genes in the 4 models, obtained the z-scored risk scores, and divided the samples with risk scores greater than zero after z-scorization into the high-risk group and those with risk scores less than zero into the low-risk group. The prognostic difference in OC between the two groups of samples was calculated. The ROC and OC KM curves of the two models are shown in Additional file 2: Figure S2B–D. The ROC curves of the two models based on the TCGA data showed a poor construction, and the AUC values at 1, 3 and 5 years were lower than the average AUC values of our 6-gene model. The KM curves of the Wang model showed that the high-risk group was significantly related to the poor prognosis of patients with OC (p < 0.01). No significant difference was observed in the prognosis between the high and low subgroups of the Sabatier model. Overall, our model is more reasonable and effective with fewer genes.

At present, effective predictive markers for immunotherapy are limited. The identification of new predictive markers is crucial for the further advancement of precision immunotherapy. Therefore, we retrieved the IMvigor210 dataset [25], which contains the gene expression profile data of PD-L1 immunotherapy metastatic urothelial carcinoma (mUC) patients, to explore whether the 6-gene model can predict the benefits of immunotherapy. The results of the KM curve showed that a higher risk score was associated with poorer survival in mUC patients (Additional file 2: Figure S2E). We used the same method to evaluate the risk score of patients and analysed the difference in the risk score under different response states, as shown in Additional file 2: Figure S2F, G. The risk score of complete response (CR)/partial response (PR) patients (responders) was significantly lower than that of progressive disease (PD)/stable disease (SD) patients (nonresponders), indicating that patients in the low-risk score group had a good response to immunotherapy. Furthermore, the KM curve revealed that a lower risk score was associated with better survival in mUC patients receiving immunotherapy (Additional file 2: Figure S2H).

### Expression of the signature genes in OC tissues

**Fig. 6:**
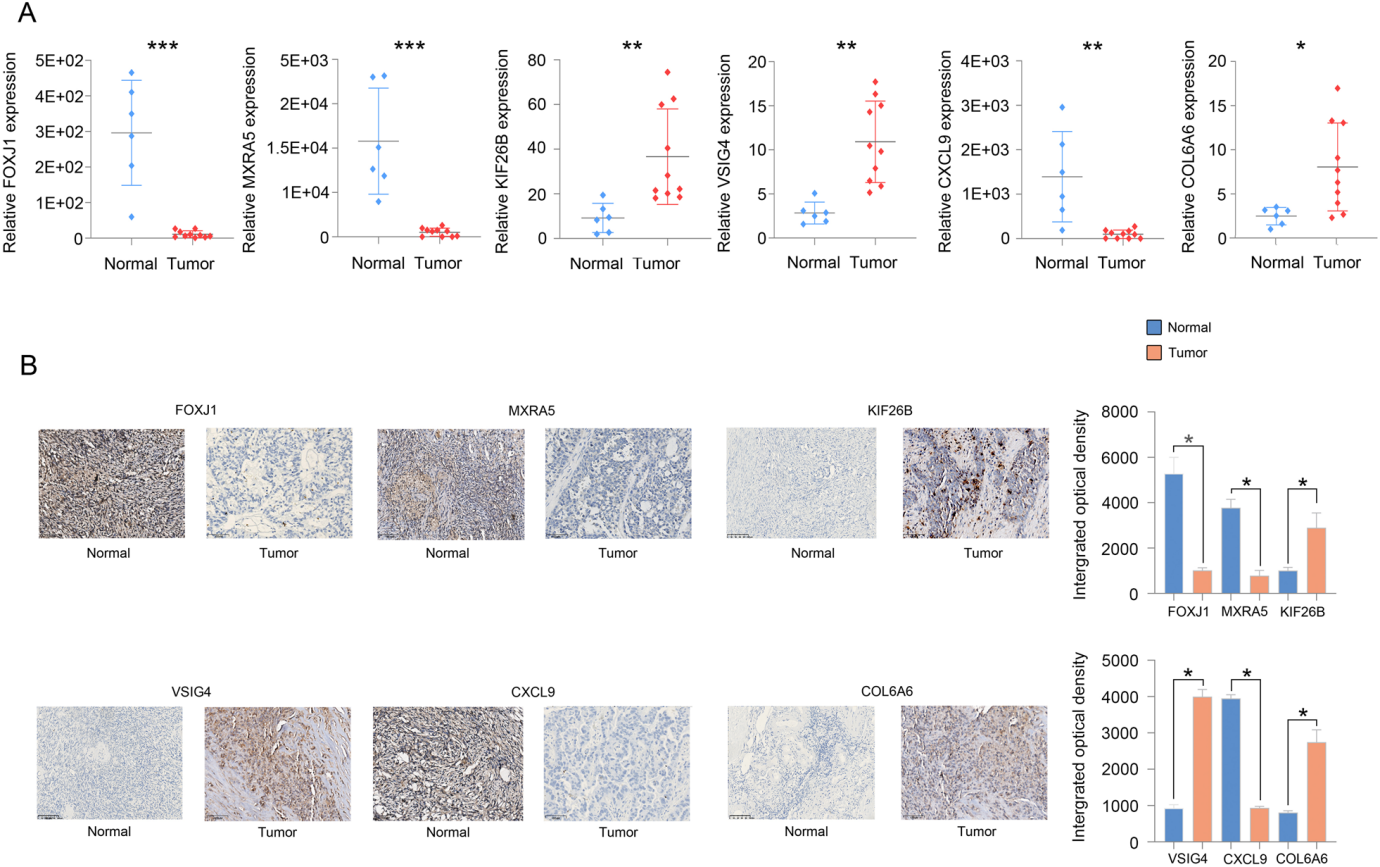
The qPCR (**A**) and IHC (**B**) results showed that FOXJ1, MXRA5 and CXCL9 expression was low and that KIF26B, VSIG4 and COL6A6 expression was high in OC tissues

Furthermore, to verify the accuracy of the 6-gene signature, we examined the expression of the signature genes (FOXJ1, MXRA5, KIF26B, VSIG4, CXCL9 and COL6A6) in clinical samples from OC patients by qPCR and IHC analyses. The qPCR and IHC results showed that the expression of FOXJ1, MXRA5 and CXCL9 was low, while that of KIF26B, VSIG4 and COL6A6 was high in OC tissues (Fig. 6A, B).

### Biological functions of the signature genes in OC cells

**Fig. 7:**
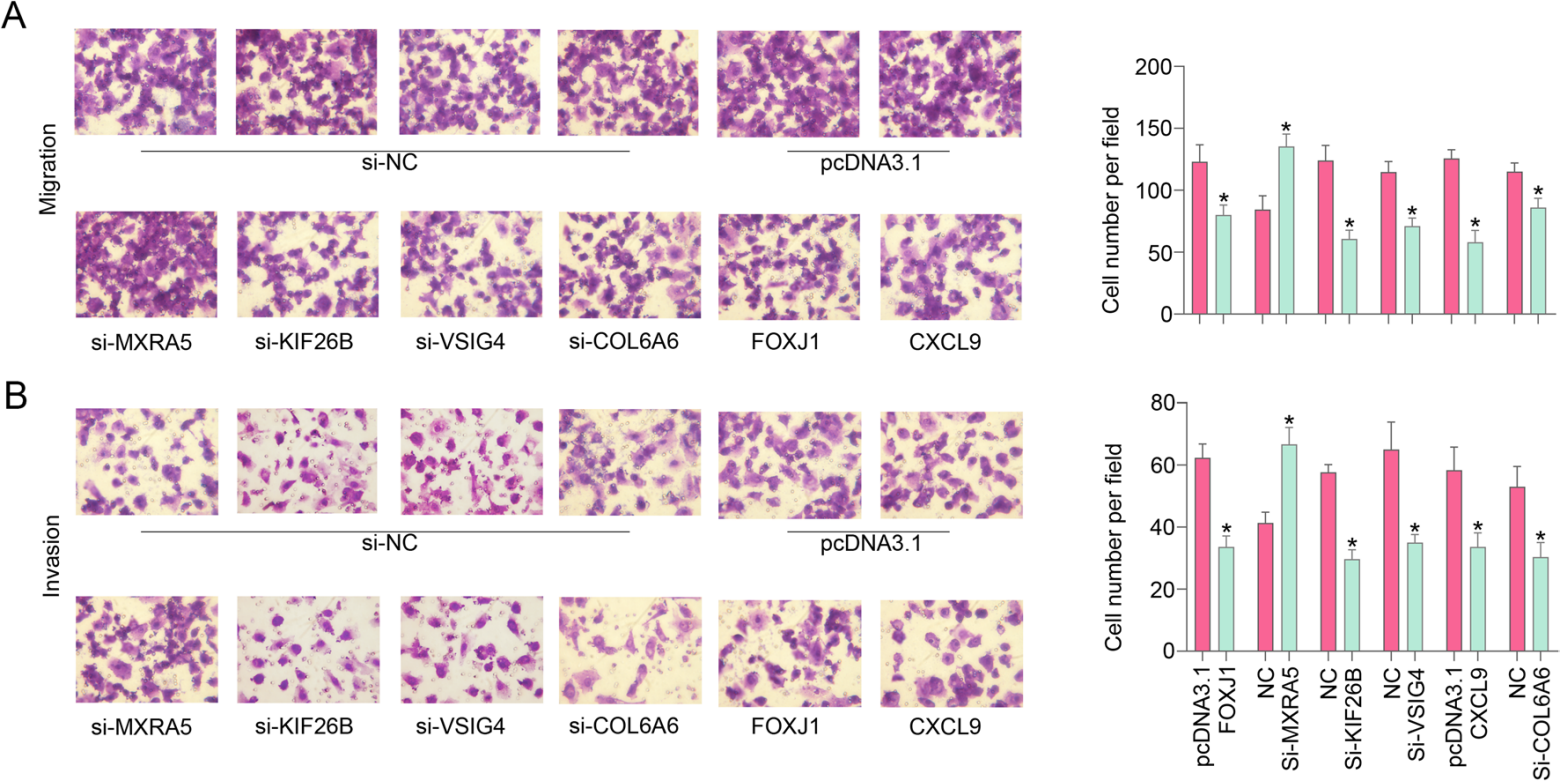
Transwell assays revealed that downregulation of KIF26B, VSIG4 or COL6A6 and upregulation of FOXJ1 or CXCL9 significantly inhibited the invasion (**A**) and migration (**B**) abilities of CAOV3 cells in vitro; downregulation of MXRA5 promoted the invasion and migration abilities of CAOV3 cells

To clarify the functional roles of the signature genes in OC cells, siRNA was used to reduce the expression of MXRA5, KIF26B, VSIG4 and COL6A6, and overexpression plasmids were used to overexpress FOXJ1 and CXCL9. For MXRA5, the transcript length was 9804 bp, which was too long to construct an overexpression plasmid and thus, Si-MXRA5 was used for functional assays. Transwell assays were used to determine the invasion and migration abilities of CAOV3 and A2780 cells in vitro, and the results showed that reduced KIF26B, VSIG4 or COL6A6 expression and FOXJ1 or CXCL9 overexpression significantly inhibited CAOV3 cell invasion and migration. Moreover, low MXRA5 expression increased the invasion and migration abilities of CAOV3 cells (Fig. 7A, B). We observed the same results in A2780 cells (Additional file 3: Figure S3) that reduced KIF26B, VSIG4 or COL6A6 expression and FOXJ1 or CXCL9 over-expression significantly inhibited invasion and migration, and low MXRA5 expression increased the invasion and migration abilities of A2780 cells.

## Discussion

Although the incidence and mortality of OC has been declining, fewer than half of women survive more than 5 years after diagnosis due to its high invasiveness, a lack of specific early symptoms and a lack of effective early detection strategies [26]. Therefore, the main challenge is to develop an accurate prognostic model to provide criteria for making clinical treatment decisions.

To solve this problem, we collected genes related to OC invasion and used gene expression data from public databases, such as TCGA and GEO, to establish molecular subtypes of OC based on tumour invasion-related genes. The relationships between molecular subtype and prognosis and clinical features were further evaluated. The prognostic risk model constructed by DEGs between OC molecular subtypes can better evaluate the prognosis of OC patients; moreover, the GEO gene expression dataset was further used to verify that the prognostic risk model has good performance. FOXJ1, MXRA5, KIF26B, VSIG4, CXCL9 and COL6A6 were screened out. High expression of KIF26B, VSIG4 and COL6A6 is related to high risk and is a risk factor for poor prognosis. High expression of FOXJ1, MXRA5 and CXCL9 is related to low risk and is protective. Moreover, the results of the qPCR and immunohistochemistry staining assays revealed that KIF26B, VSIG4 and COL6A6 were upregulated and that FOXJ1, MXRA5 and CXCL9 were downregulated in OC tissues. The functional study showed that the expression of KIF26B, VSIG4, COL6A6, FOXJ1, MXRA5 and CXCL9 can regulate the migration and invasion abilities of OC cells. Our signature is more accurate than others, and we established a more reasonable and efficient model with fewer genes.

FOXJ1 is a member of the FOX family and has been reported to be involved in tumorigenesis. Single-cell sequencing of endometrial and ovarian tumours has shown that FOXJ1 is expressed in endometrial tumours and is positively correlated with the disease specificity and overall survival of endometrial cancer patients [27]. Knockdown of FOXJ1 inhibits proliferation, migration, invasion and glycolysis in laryngeal squamous cell carcinoma cells [28]. Decreased FOXJ1 expression and cilia formation in ependymomas and choroid plexus tumours are markers of poor prognosis and are therefore useful biomarkers for evaluating these tumours [29]. Patients with malignant pleural mesothelioma and a high MXRA5 mutation frequency have a longer survival time [30]. However, in glioblastoma, MXRA5 mutations do not significantly alter prognosis [31]. This suggests that MXRA5 plays different roles in different tumours. KIF26B is a downstream target of the zinc finger protein Sall1, which is involved in the development of various tumours. Studies have shown that high KIF26B expression in colorectal cancer tissues can increase the proliferation, migration, and invasiveness of colorectal cancer cells and increase tumour depth and lymph node metastasis and that high KIF26B expression is closely related to poor prognosis [32]. Increased expression of KIF26B in gastric cancer is associated with tumour size, lymph node metastasis positivity or distant metastasis and poor prognosis [33]. KIF26B also promotes the development and progression of breast cancer and plays a key role in breast cancer growth and metastasis [34, 35]. Downregulation of KIF26B inhibits the viability, proliferation and invasiveness of hepatocellular carcinoma cells [36]. VSIG4 is a newly discovered immune checkpoint of the B7 family of immunoregulatory proteins. Recent studies have shown that VSIG4 overexpression in advanced gastric cancer is independently and strongly associated with poor prognosis, which indicates its potential as an important prognostic indicator in advanced gastric cancer [37]. Serum VSIG4 levels were found to be significantly increased in patients with lymphoma and can be used for lymphoma diagnosis [38]. In clear cell renal cell carcinoma, VSIG4 is upregulated and indicates a poor prognosis [39]. The chemokine CXCL9 plays an important role in the inflammatory process and angiogenesis and is also related to the occurrence, development and metastasis of tumours. CXCL9 is highly correlated with CD8 + T cells and natural killer (NK) cells in colorectal cancer and has an antitumour immune effect [40]. In bladder cancer, CXCL9 expression is negatively correlated with the recurrence rate, and high CXCL9 expression can improve the prognosis of bladder cancer patients [41]. Increased CXCL9 expression in gastric cancer leads to decreased proliferation and metastasis of gastric cancer cells [42]. COL6A6 is a beaded fibrocollagen located near the basement membrane and is involved in maintaining epithelial and endothelial cell transformation [43]. COL6A6 upregulation significantly inhibits the proliferation, invasiveness, migration and epithelial-mesenchymal transition (EMT) of pituitary tumour cells and significantly inhibits tumour size, tumour volume and tumour weight [44].

However, a limitation of this study is that some data lacked clinical follow-up information, and further genetic and experimental studies along with experimental verification are needed with larger sample sizes. Moreover, direct clinical application tests of this prognostic model will need to be conducted. We will further verify the predictive ability of the gene signature with clinical samples in a follow-up study. This study proposes that the prediction model based on six genes is applicable, and the risk score, which can be calculated through an algorithm, can be used for risk classification prediction.

In summary, we established an invasion-related 6-gene signature to evaluate the prognosis of OC patients. Furthermore, to verify the signature, we used the TCGA-test and GSE32062 and GSE17260 chip datasets for testing and finally obtained a good risk prediction effect in those datasets. Moreover, the results of the qPCR and immunohistochemistry staining assays revealed that KIF26B, VSIG4 and COL6A6 were upregulated and that FOXJ1, MXRA5 and CXCL9 were downregulated in OC tissues. The functional study showed that the expression of KIF26B, VSIG4, COL6A6, FOXJ1, MXRA5 and CXCL9 can regulate the migration and invasion abilities of OC cells. These results suggest that the signature could potentially be used to evaluate the prognostic risk of OC patients.

## Supplementary Information

**Additional file 1.** KEGG pathway and GO functional enrichment analyses on the DEGs

**Additional file 2.** Prognostic analysis of the risk model and clinical features

**Additional file 3.** Transwell assays were used of A2780 cells

**Additional file 4.** Invasion-related gene sets

**Additional file 5: Table S2.** The clinicopathological characteristics of ovarian cancer tissues

**Additional file 6.** siRNA sequences and primers for qRT-PCR analysis

**Additional file 7.** 384 upregulated genes and 9 downregulated genes

**Additional file 8.** The detailed information of Go

**Additional file 9.** The detailed information of KEGG

**Additional file 10.** 7 prognosis genes

## Data Availability

The data and materials can be obtained from the first author and corresponding author.
