# Supplementary figures and images for: Establishment and validation of a novel invasion-related gene signature for predicting the prognosis of ovarian cancer
Source: Cancer Cell Int. 2022 Mar 15;22:118. doi: 10.1186/s12935-022-02502-4 (PMC8922755; doi:10.1186/s12935-022-02502-4)

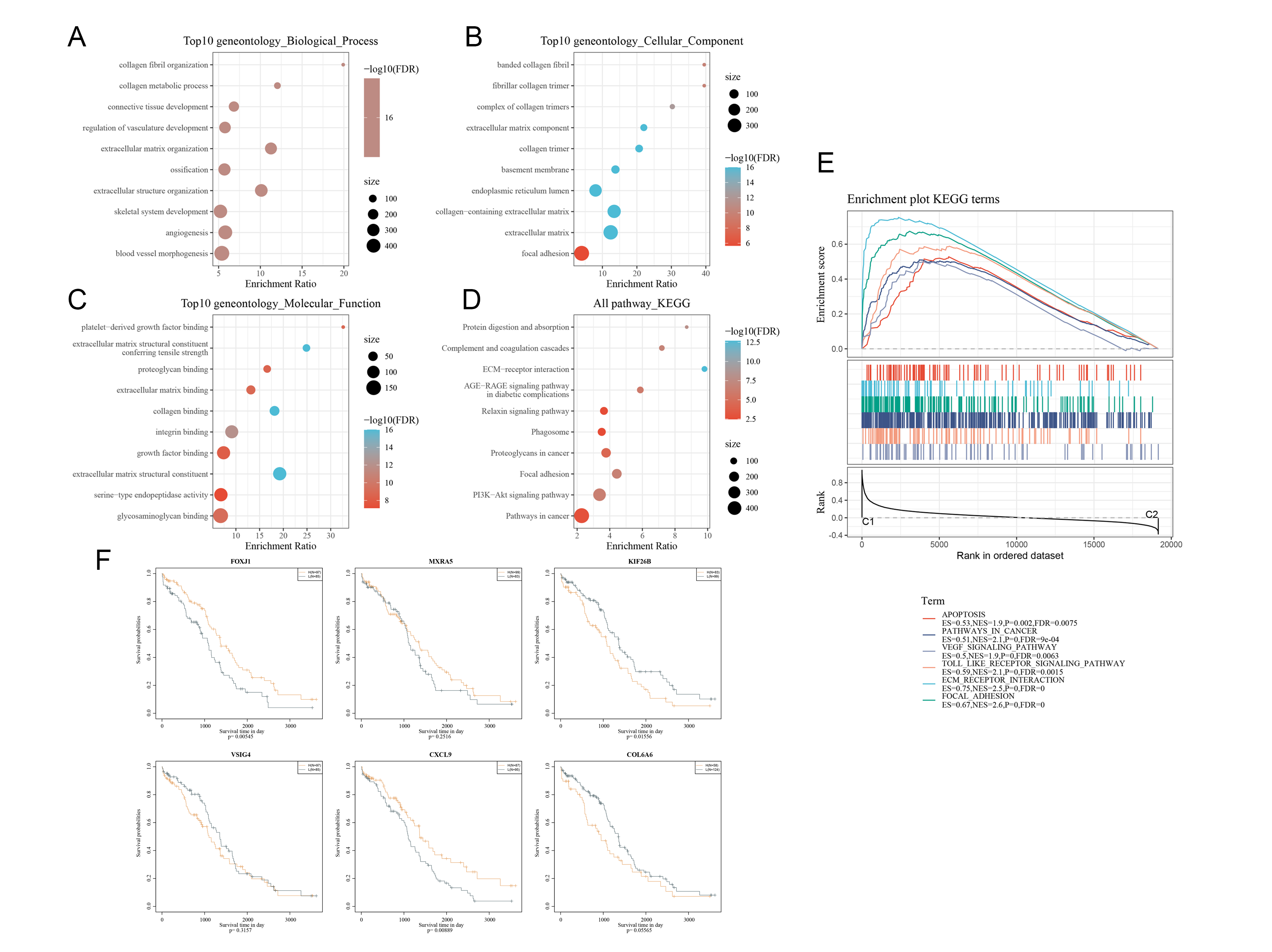

Supplement: Supplementary file 1 — Additional file 1. KEGG pathway and GO functional enrichment analyses on the DEGs [file 12935_2022_2502_MOESM1_ESM.png]

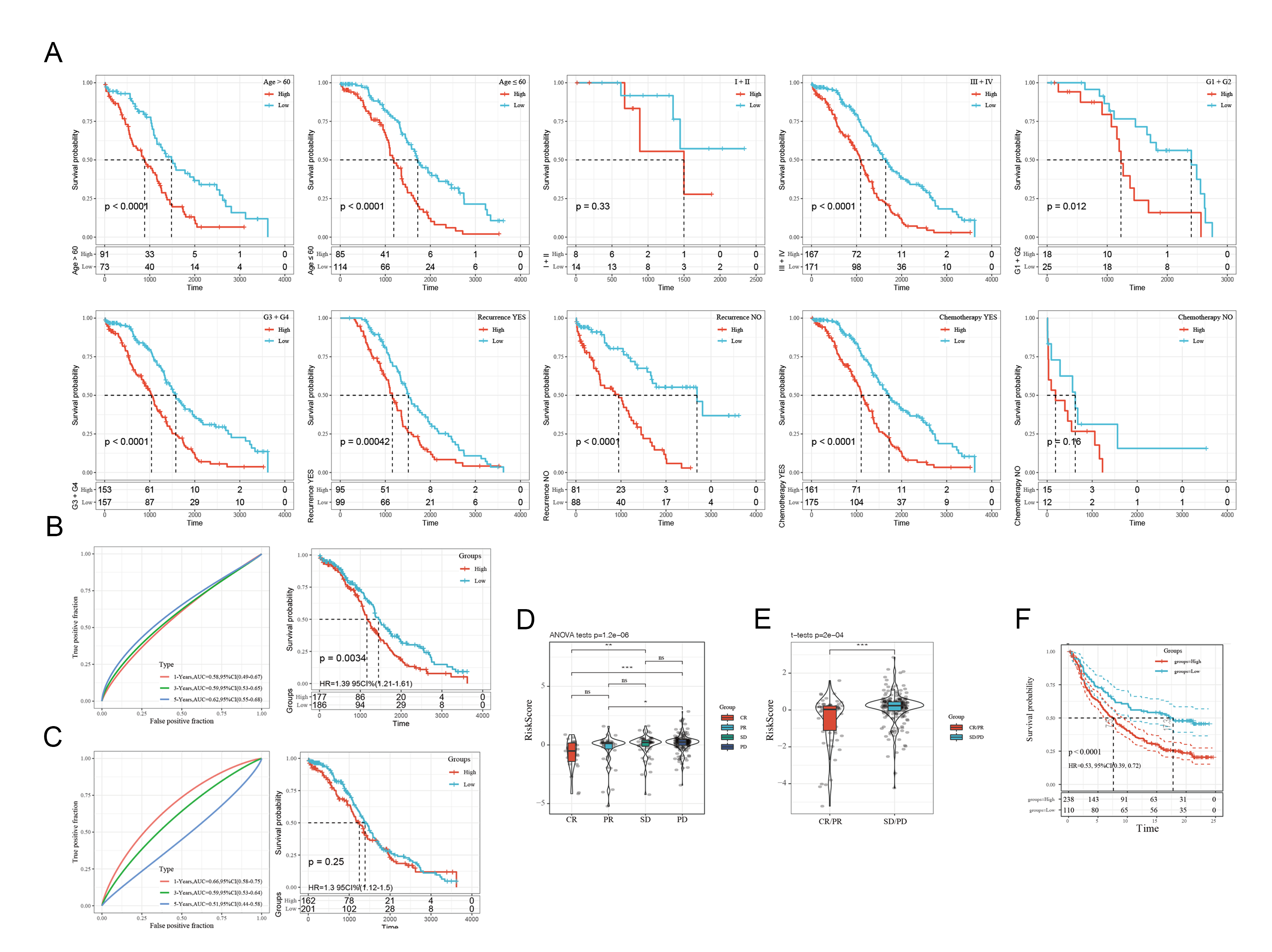

Supplement: Supplementary file 2 — Additional file 2. Prognostic analysis of the risk model and clinical features [file 12935_2022_2502_MOESM2_ESM.png]

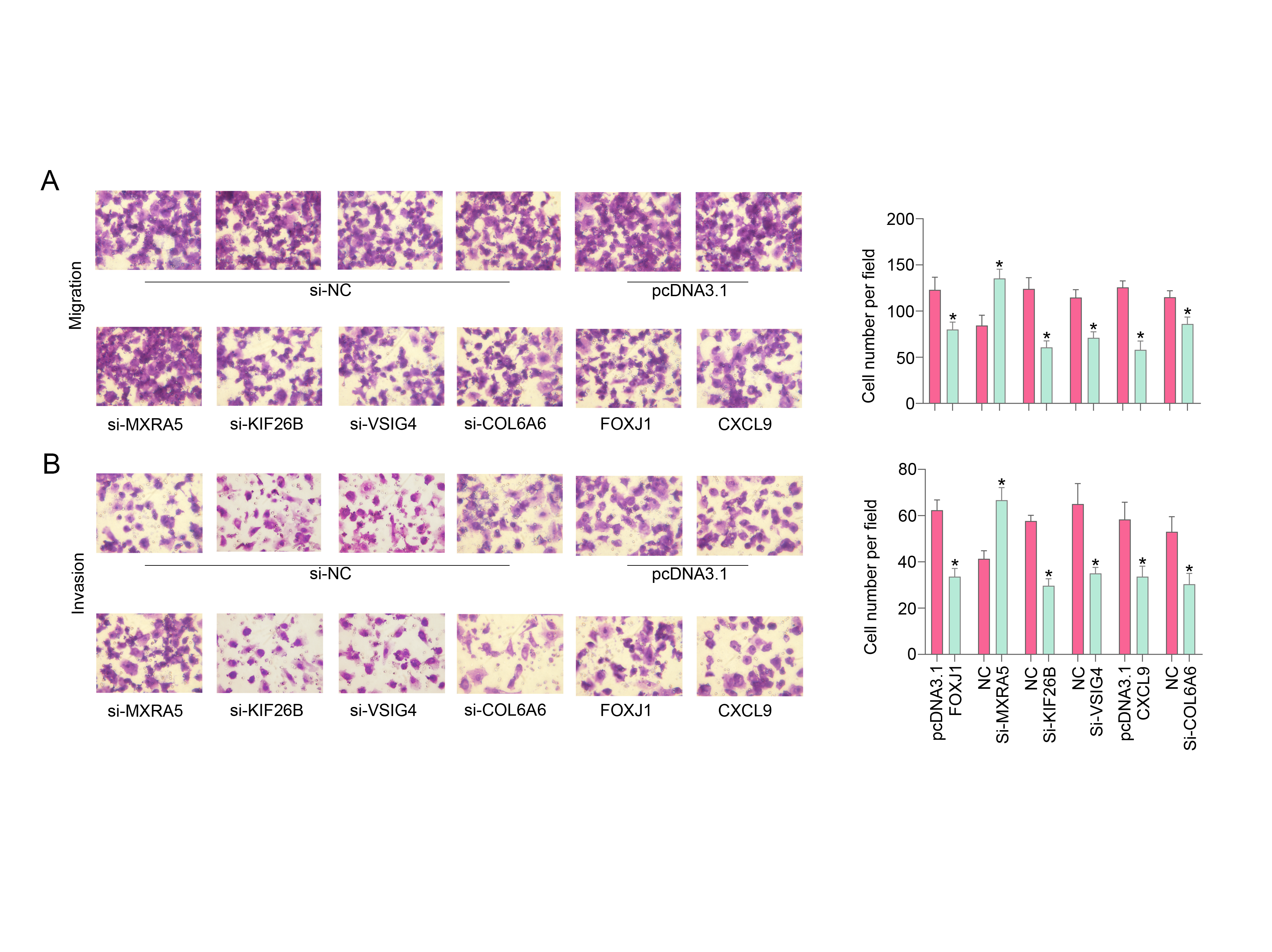

Supplement: Supplementary file 3 — Additional file 3. Transwell assays were used of A2780 cells [file 12935_2022_2502_MOESM3_ESM.png]
