# Supplementary material for: Establishment and validation of a novel invasion-related gene signature for predicting the prognosis of ovarian cancer
Source: Cancer Cell Int. 2022 Mar 15;22:118. doi: 10.1186/s12935-022-02502-4 (PMC8922755; doi:10.1186/s12935-022-02502-4)
Supplement: Supplementary file 5 — Additional file 5: Table S2. The clinicopathological characteristics of ovarian cancer tissues [file 12935_2022_2502_MOESM5_ESM.docx]

| N | |
| --- | --- |
|  |  |
| Age (yeas) |  |
| < 50 | 2 |
| ≥ 50 | 8 |
| Gender |  |
| Female | 10 |
| FIGO stage |  |
| I-II | 0 |
| III-IV | 10 |
| Type of ovarian cancer |  |
| Adenocarcinoma | 10 |
|  |  |
|  |  |
|  |  |

Table The clinicopathological characteristics of ovarian cancer
