# Supplementary material for: Establishment and validation of a novel invasion-related gene signature for predicting the prognosis of ovarian cancer
Source: Cancer Cell Int. 2022 Mar 15;22:118. doi: 10.1186/s12935-022-02502-4 (PMC8922755; doi:10.1186/s12935-022-02502-4)
Supplement: Supplementary file 6 — Additional file 6. siRNA sequences and primers for qRT-PCR analysis [file 12935_2022_2502_MOESM6_ESM.docx]

| Si-MXRA5 | 5’-GAUAUUAGAUUUCCUUGUATTUACAAGGAAAUCUAAUCUCGC-3’ |
| --- | --- |
| Si-KIF26B | 5’-GCUGUGAUUCACGACAAACTTGUUUGUCGUGAAUCACAGCCG-3’ |
| Si-VSIG4 | 5’-AGAAACUCUCUGUCUCCAATTUUGGAGACAGAGGUUUCUGG-3’ |
| Si-COL6A6 | 5’-CAUGGGUGGCAGUACUUAUTTAUAAGUACUGCCACCCAUGGC-3’ |
| FOXJ1  Forward | 5’-ACTCGTATGCCACGCTCATCTG-3’ |
| FOXJ1  Reverse | 5’-GAGACAGGTTGTGGCGGATTGA-3’ |
| MXRA5  Forward | 5’-TCACCGCTGAGACAGACACTGT-3’ |
| MXRA5  Reverse | 5’-CCGTTGTATCCTGGTATTCGGAG-3’ |
| KIF26B  Forward | 5’-AGAGGTGATCCAGTCTGTGGTC-3’ |
| KIF26B  Reverse | 5’-GGAATGATGCCCAGGTTCTGCA-3’ |
| VSIG4  Forward | 5’-GATGGCAACCAAGTCGTGAGAG-3’ |
| VSIG4  Reverse | 5’-CCTGGCATTGAAGGCTAATCCTC-3’ |
| CXCL9  Forward | 5’-CTGTTCCTGCATCAGCACCAAC-3’ |
| CXCL9  Reverse | 5’-TGAACTCCATTCTTCAGTGTAGCA-3’ |
| COL6A6  Forward | 5’-GGATCGTTCGCAACATCTGTACC-3’ |
| COL6A6  Reverse | 5’-GCTGACCTTCAAGCAAAGTCTGC-3’ |
| GAPDH  Forward | 5’-CAGCCTCAAGATCATCAGCA-3’ |
| GAPDH  Reverse | 5’-TGTGGTCATGAGTCCTTCCA-3’ |

Supplementary Table 2. siRNA sequences and primers for qRT-PCR analysis.
